# Developing and Sustaining Recovery-Orientation in Mental Health Practice: Experiences of Occupational Therapists

**DOI:** 10.1155/2017/5190901

**Published:** 2017-02-26

**Authors:** Alexandra Nugent, Nicola Hancock, Anne Honey

**Affiliations:** The University of Sydney, Cumberland Campus, 74 East St., Lidcombe, NSW 2141, Australia

## Abstract

**Background/Aim:**

Internationally, mental health policy requires clinicians to shift from a medical to a recovery-oriented approach. However, there is a significant lag in the translation of policy into practice. Occupational therapists have been identified as ideally situated to be recovery-oriented yet limited research exploring how they do this exists. This study aimed to explore Australian occupational therapists' experiences of developing and sustaining recovery-orientation in mental health practice.

**Methods:**

Semistructured, in-depth interviews were conducted with twelve occupational therapists working across different mental health service types. Participants identified themselves as being recovery-oriented. Data were analysed using constant comparative analysis.

**Results:**

Occupational therapists described recovery-oriented practice as an active, ongoing, and intentional process of seeking out knowledge, finding fit between understandings of recovery-oriented practice and their professional identity, holding hope, and developing confidence through clinical reasoning. Human and systemic aspects of therapists' workplace environment influenced this process.

**Conclusions:**

Being a recovery-oriented occupational therapist requires more than merely accepting a specific framework. It requires commitment and ongoing work to develop and sustain recovery-orientation. Occupational therapists are called to extend current leadership activity beyond their workplace and to advocate for broader systemic change.

## 1. Introduction

In contrast to a medical understanding of mental health recovery as “cure,” recovery as defined by people with lived experience of mental illness refers to an individual's ongoing and nonlinear journey towards creating and living “a meaningful and contributing life in a community of choice with or without the presence of mental health issues” [1]. With this definition of recovery, people using mental health services, or “consumers,” are necessarily active and leading agents in their own recovery [2, 3]. Therefore, recovery cannot be done to, or for, consumers by service providers [4]. Rather, services are called on to adopt recovery-oriented practice to better support consumers as they take the lead in their unique recovery journeys [5–7].

Recovery-oriented practice involves being consumer-focused, facilitating choice and opportunity, and holding and promoting hope for each consumer's future [8]. Recovery journeys are driven by the consumers and their choices, goals, and rights [9]. This requires movement away from medically oriented care in which absence of symptoms is the primary outcome measured and medication is the focus of treatment [9]. Recovery-oriented practice instead strives to enable consumers to reclaim and develop new meaning in their lives [8, 10].

Occupational therapists have repeatedly been identified as ideally placed to facilitate a shift towards recovery-oriented practice due to the congruence between occupational therapy theory and recovery-oriented principles [11–14]. Autonomy, choice, strong therapeutic partnerships, and enablement are central components of both client-centred occupational therapy [15] and recovery-oriented practice.

Current policies and guidelines across the English-speaking world, including Australia, require the adoption of recovery-oriented practice in mental health service delivery [16–20]. Further, recent outcome studies indicate that recovery-oriented practice may lead to better consumer engagement in treatment and quality of life [21] and that aspects of recovery-oriented practice such as choice and therapeutic alliance are strongly linked to positive recovery outcomes [22]. Yet, commentators in the field have consistently identified a lag in the implementation of recovery-oriented practice in mental health services (e.g., [23]). In fact, services have been described as “simply repackaging old wine in the new bottle of recovery language” [24].

Research that, with the exception of nursing, does not distinguish between professional backgrounds has identified a range of challenges in operationalising recovery-oriented practice (e.g., [25, 26]). These include a lack of clarity regarding what recovery-oriented practice entails [7, 8, 27], staff scepticism [27, 28], and incongruence between recovery and workplace priorities [7, 25–27]. Facilitators of recovery-oriented practice are less commonly reported but include developing a workplace culture that values innovation and clinicians having a willingness to test the boundaries set by restrictive organisations [25, 27, 29]. Various guidelines are available which explain what recovery-orientation looks like in practice (e.g., [9]). However, missing from the literature are (1) a nuanced understanding of how recovery-oriented clinicians actually go about sustaining their recovery-orientation within the context of the identified barriers and facilitators and (2) a specific investigation of recovery-orientation amongst occupational therapists despite assertions that they are uniquely placed to be recovery-oriented.

The aim of the current study was to explore Australian occupational therapists' experiences of developing and sustaining recovery-orientation in mental health practice.

## 2. Methods

### 2.1. Study Design

A detailed and contextualised understanding of the experiences of occupational therapists is required to aid in the operationalisation of recovery-oriented approaches. Therefore, a qualitative design was needed [30]. Grounded theory data collection and analysis techniques were used to ensure methodological rigour and inductively explore and understand participants' experiences. The techniques used were purposive and theoretical sampling, concurrent data collection and analysis, and constant comparative analysis [31]. These are described below. Ethical approval for this research was obtained from the University of Sydney Human Research Ethics Committee.

### 2.2. Initial Sampling and Recruitment

Participants were purposively sampled based on their direct experience with, and thus insight into, the research topic. Australian occupational therapists working in any mental health service who identified themselves as adopting recovery-oriented practice were eligible to participate. Participants were recruited through an email sent to members by the Mental Health Interest Group in the NSW Division of Occupational Therapy Australia. All participants provided written informed consent.

### 2.3. Data Collection and Analysis

Data collection and analysis were conducted concurrently to allow the researchers to pursue lines of inquiry informed by their ongoing analyses [30]. Data were collected through in-depth, semistructured interviews to foster a deep understanding of participants' unique experiences [30]. An interview guide was designed to encourage participants to raise important issues within the research topic and allow the interviewer to flexibly follow up on these issues [32]. Participants were asked about what being recovery-oriented meant for their own practice and how they sustained their recovery-orientation in their unique context. Because preliminary analysis of each interview occurred before engaging with the next participant [31], questions could be modified in response to emerging ideas. For example, after four interviews, risk management was identified as an important concept. Risk was discussed with subsequent participants to deepen the researchers' understanding. Interviews lasted 40–60 minutes and were audio recorded and transcribed verbatim.

Constant comparative analysis was used throughout the study [31]. Each section of data was coded with one or more words or phrases that described its underlying meaning. For example, the statements “not all managers have an understanding of what recovery-oriented practice might look like” and “it's hard if you have to be the one to convince your boss” were coded “lack of understanding from seniors.” These initial codes were compared with each other and with new data as they were collected. Conceptually similar codes were grouped to form categories [31]. The broad category “workplace attitudes and culture,” for example, was created by comparing the initial code “lack of understanding from seniors” with other initial codes such as “negative team attitudes.” Methodological rigour was enhanced through frequent reflective discussions between authors about the codes and their relationships. This enabled consensus to be reached on the interpretation of data.

After analysis of twelve interviews, it became apparent that while no new codes were emerging, two potentially important concepts were underdeveloped: the fit between occupational therapy and recovery-oriented principles and the ongoing development (as opposed to simply maintenance) of recovery-orientation. Therefore, in accordance with theoretical sampling, further data collection was required. Examination of transcripts revealed that some participants had not been asked to expand on these issues as they had emerged in later interviews. Thus, follow-up interviews were conducted with a purposively selected subset of existing participants using a new interview guide designed to address these topics. Participants whose interviews had not covered these topics in detail were selected to obtain coverage of the topic across different service settings and levels of seniority [33]. After five follow-up interviews, new data were no longer revealing new concepts and all categories were well covered. Data collection therefore ceased, with no recruitment of new participants being required [30]. Participant characteristics are provided in Table 1.

In the final stage of constant comparative analysis, the relationships between categories were confirmed, and a framework was developed to explain the experiences of occupational therapists developing and sustaining recovery-orientation in mental health practice.

## 3. Results

Participants described developing and sustaining recovery-oriented practice as an active and ongoing process. It was not a static, singular decision to be recovery-oriented or not. Neither was it a destination that participants had reached or hoped to reach. Rather, participants relayed stories of continued growth and a process or journey of striving towards recovery-oriented practice. This journey was shaped by participants' environments and they described both working within and attempting to modify or change their environments to enhance their capacity to work in a more recovery-oriented manner.

### 3.1. A Journey of Actively Striving towards Recovery-Oriented Practice

Participants described a need to continuously develop and strengthen their recovery-orientation. In order to do this, they detailed an active and ongoing process consisting of four components: intentionally seeking out knowledge, working to find fit or synergy between recovery-oriented practice principles and their professional identity and roles, maintaining hope, and developing confidence in defending recovery-oriented practices by strengthening their clinical reasoning skills.

#### 3.1.1. Seeking Out Knowledge

All participants emphasised the importance of having a clear conceptualisation of recovery and recovery-oriented practice. Cultivating this was an intentional and active process. Participant understandings were constantly developing in response to new experiences and knowledge. They all highlighted a need to seek out opportunities to develop their knowledge, including learning directly from people with lived experience of mental illness. Casey highlighted the importance of taking time to “actually hear first-hand people's perspectives.” In addition, participants described seeking out and reading recovery-based literature and personal narratives, attending recovery-focused training and conferences, and seeking advice from other recovery-oriented clinicians. Charlotte advised: “Do the course! Start doing workshops… things that are recovery-focused… you've got to have an understanding of what it means… It took me a lot of conscious effort.”

#### 3.1.2. Finding Fit

As participants learnt more and more about recovery and recovery-oriented practice, they described simultaneously integrating their developing conceptualisations with their personal and professional identities. All participants described a strong compatibility or synergy between recovery-oriented principles and occupational therapy philosophies. They talked about aspects such as person-centredness, supporting engagement in meaningful activity, empowerment of the individual, and taking a holistic and contextualised approach as being key and common facets of both recovery-oriented practice and occupational therapy. For example, Pat said, “I think naturally [occupational therapists are] attuned to being… holistic in our approach… the importance of meaningful activity … how the environment plays a part in people's recovery… people developing meaningful roles… It's ingrained in us in our training.” Similarly, Jamie felt that recovery-oriented practice “ties in really well with OT… finding out what is meaningful to them and framing our engagement with them around that,” while Chris described the core business of occupational therapy as being “to facilitate them to have the life that they want, where they want.”

Four participants suggested that recovery-orientation was more compatible with occupational therapy than other health professions. For example, Chris believed that occupational therapy was “a lot more about a person's roles and their overall functioning and how their entire life is, rather than ‘you have this exact symptom, this is how you fix it.'” Because of this synergy, some participants saw the shift to recovery-oriented practice as an opportunity for the profession: “What's exciting about… recovery-orientated practice [is that it] supports a lot of what occupational therapists do… It might get to a point where… OTs find it easier to practice [and other professions] value the role of OT more” (Casey).

While two participants saw the alignment between occupational therapy and recovery as a straightforward “marriage made in heaven” (Charlotte), most also identified areas of potential misfit or challenge: “I still find ‘recovery' a hard concept… trying to figure out what it means for me personally [and] professionally… What service it is that I want to be involved in providing… That was the first battle” (Ava).

Therapists described grappling to find fit between recovery and elements of their occupational therapy learning and identity. At times they needed to actively change their ways of thinking and “unlearn” old, medically oriented understandings and practices. Charlotte explained, “It took me a while… to move away from [a] deficit focus… When I went to uni it… looked at people's deficits… A lot of the assessments we still use are deficit-focused.” Participants also wrestled with relinquishing the role of expert clinician to offer choice and hand control over to the consumer. For example, Chris thought that health professionals were trained to use their clinical expertise and to expect consumers to “kneel at the feet of clinicians.” Likewise, Addison described her struggle with “trying to drop the therapist and the ‘solving problems' part,” adding that “sometimes it's not about solving the problem… it's about walking the journey.”

Finally, while being not identified by participants as a challenge, a number appeared to define recovery through an occupational therapy or clinically based framework, emphasising independence and function as desired outcomes. For example, Amelia described recovery as being about consumers becoming “independent and able to look after themselves… in the community,” and Chloe stated that having “that end goal of independence where possible is probably the tick we [OTs] have in our box compared to others.”

#### 3.1.3. Maintaining Hope

Having knowledge of recovery and recovery-oriented practice and being able to find fit between emerging recovery knowledge and occupational therapy were not enough for participants to practice in a recovery-oriented manner. They also stressed the importance of having and maintaining hope and holding a genuine belief in recovery. Casey explained, “You have to [have] hope that… recovery is possible… It would become an awful place to work if you thought that was as good as it gets for people.”

A belief that people can recover drove participants' recovery-orientation. Jessica, for example, described needing to have “a really strong belief that, [for] every single consumer that I work with… this is not your life. It will get better than this.” Being witness to people recovering and seeing consumers benefiting from a recovery-oriented approach helped to ignite participants' hope and encouraged them to further pursue this approach to practice.

I was [initially] a bit nervous… these people have been through the mental health service for such a long period of time, so how much change are we going to be able to achieve? It really has amazed me… If you're working under a recovery model it is possible to make change. (Pat)

In order to actively nurture this hope, some therapists sought connections with people recovering from mental illness outside of their immediate workplaces. For example, because Jessica's work primarily involved contact with people in acute phases of illness, she volunteered at the Hearing Voices Network in order to “spend time with people who are recovered and that maintains my hope.” Casey also described observing people living their lives beyond the immediate workplace as critical because “if you look at the bigger picture, you can be… more hopeful.”

#### 3.1.4. Developing Confidence through Clinical Reasoning

To apply their knowledge and adopt practices they believed in, participants also described needing to develop confidence in their ability to make sound clinical decisions and to justify recovery-oriented practices when they conflicted with practice-as-normal. They particularly highlighted the practices around positive risk taking, a central mantra of recovery-oriented practice. Participants repeatedly talked about the confidence they needed to support and advocate for each consumer's right to choose to take positive risks. For example, Chris noted that being able to help consumers to take risks with treatments that might benefit their recovery “only comes with the confidence that your clinical skills and your clinical reasoning and decision making… are all good enough.” Similarly, Jessica explained that because “the support network isn't really there yet to help us make those ‘risky decisions'… you have to have really, really strong clinical reasoning and understanding of recovery to be able to justify why you made those decisions.”

As they gained more experience and developed sound clinical reasoning skills, clinicians described becoming more comfortable and confident with advocating for recovery-oriented practice. Chris summed this up by saying that “the more of a new grad you are, the less comfortable you feel implementing recovery practices because it involves risk… The less experienced you are, the more frightening that is.”

### 3.2. Working on and Working within the Environment

The journey of striving towards recovery-oriented practice was inextricably linked to and influenced by participants' work environments. Participants reported navigating, utilising, and, at times, struggling with both human and structural aspects of their environments in order to develop and sustain their recovery-orientation to practice.

#### 3.2.1. Human Aspects

The human environment of the workplace, consisting of coworkers, supervisors or leaders, and peer workers, both positively and negatively influenced participants' ability to develop and sustain recovery-orientation. Most participants reported currently or previously experiencing negative coworker attitudes towards recovery. Medical orientation, poor communication, and a general lack of hope thwarted participants' recovery-oriented growth and discouraged their belief in recovery-oriented practice. Ava, describing a previous workplace, said the following:

[Negative attitudes] can really give you a negative point of view of your professional development and your career. Like… there's no point in trying to improve or change things because these people have been around longer than you and have kind of been damaged by being burnt out or not knowing the latest evidence or concepts.

Casey explained how some staff attitudes in a forensic context made it difficult to be recovery-oriented: “they still want to see people punished and that can be really awful… they want to contain these people in these settings. That's a real challenge.”

In contrast, working with a team of like-minded, recovery-oriented coworkers was perceived as highly beneficial. “You need to be strongly supported by a really good and caring team of people… in an environment where you never feel afraid to ask a question or… to get people to check that your reasoning is sound” (Chris). In these supportive environments, participants described pursuing and using the advice and support of recovery-oriented colleagues, more senior clinicians, and supervisors. Amelia described how her recovery-oriented colleagues helped and inspired her: “Sharing of that knowledge and supporting each other… That's what makes you come in and try, even though you've been butting your head.” Addison used her sessions with her recovery-focused supervisor to “chat through… how I best could approach some situations.”

While colleagues were an important part of the human environment, decision-makers or “people at the top” who supported recovery-oriented initiatives were seen as central to a supportive workplace. Describing the benefit of recovery-focused management or decision-makers, Jordan explained that their “support flows down into the frontline staff… It's reassuring and supportive.” Casey concurred:

There is a hierarchy and if your consultant is recovery-orientated it makes a world of difference… they still direct treatments and they can push for recovery-orientated practices and have more authority than any other clinician… So, having them on board with recovery is the biggest enabler of all.

Participants also repeatedly highlighted the benefit of having people with lived experience working in their service. Peer workers were viewed as valuable assets. In Chris's workplace, for example, peer workers “are getting more involved with actually improving the [recovery focus of the] service.” Participants actively used their peer workers as sounding boards for their ideas and peer workers sometimes challenged clinicians' decision-making, prompting participants to critically reflect on their recovery-oriented principles and take action accordingly. Participants described peer workers as inspiring hope in consumers and clinicians alike. As Chloe reported, their presence “really helps with that understanding and that recovery-base and providing people hope that you can recover.”

Importantly, participants did not see their relationship with the human environment of their workplace as a one-way transaction. Therapists also described taking action to enrich their workplace culture through leadership and example. As a supervisor, Casey said that “you have to be recovery-orientated yourself… be aware of what recovery is and bring that into the conversation… be aware of what tools are available… [to] guide people to be recovery-orientated.”

Amelia suggested that, to influence the human environment, “you can use the language, you can talk about the frameworks, you can lead by example.” She saw this leadership as an obligation for occupational therapists because of the synergy between the profession's philosophies and recovery-oriented approaches: “We are the ones that need to be leading the recovery-orientated approach within health services and if we're not there is something that is really wrong.”

#### 3.2.2. Structural Aspects

The structural elements of participants' work environments presented challenges to their journey of striving towards recovery-orientation. These structural aspects were both workplace specific and generic to the mental health system more broadly. Structural challenges included the biomedical outcome-driven and risk-averse nature of the mental health system, restrictive environments, and time limitations.

First, participants described a clinical outcome-driven system that did not recognise or place value on recovery-based practices or outcomes. As Ava said, “Meeting your KPIs [key performance indicators] as an OT does not necessarily mean giving them holistic care. But if you don't meet your KPIs then to your employer you're not doing your job.”

Second, legislative and policy requirements, such as the Mental Health Act 2007 (NSW), lead to practices that restrict consumers' choice such as involuntary admission to hospital and compulsory treatment under community treatment orders (CTOs). Participants viewed these as restrictive, coercive, risk-averse, and therefore incompatible with recovery-oriented practice. Chris highlighted the challenge:

How do we actually implement [recovery-oriented practice] when we are dealing with CTOs and scheduling people… [and] having to dictate to people that they have to do these things under their forensic order… How do we… marry all those things up?

Some environments, such as acute inpatient and forensic services, were described as particularly restrictive as they reduced opportunities for consumer choice and created greater power differentials between consumers and hospital staff. Jordan, for example, described the built environment of the hospital and its swipe-card access and exit for staff as reinforcing a “don't mess with authority” message.

Finally, participants across all service settings highlighted time limitations impacting negatively their ability to sustain recovery-orientation. For community-based participants, time restraints related to caseload size. Pat explained how care coordinators with “extraordinary numbers of consumers” had difficulty getting beyond “putting out fires.” This made it difficult for them to focus on recovery. Anna described how, in acute settings, rapid consumer turnover created time constraints: “You really have no time to build up that [rapport]. They load up the patient with tablets and off [they] go.” In contrast, participants in forensic settings described the difficulty of maintaining hope and progress towards recovery goals with patients who remained in inpatient settings for many years: “People want to set recovery goals and work towards them but then feel let down by the system, so we're not actually supporting their recovery. The length of time is too much” (Casey).

Despite these challenges, all participants continued to strive towards recovery-oriented practice because “engagement with [consumers] becomes much more meaningful” (Jamie), and as Jessica said, “for me, it's pleasurable to work in a recovery-orientated way.”

## 4. Discussion

This study is, to our knowledge, the first to explore the ways in which occupational therapists go about developing and sustaining recovery-orientation in mental health practice. While it confirms previous findings on challenges and facilitators to recovery-oriented practice, it adds to the existing literature by outlining the ongoing, active, and intentional journey of striving to be recovery-oriented. Rather than simply putting their recovery-oriented beliefs into action, all participants, irrespective of workplace setting, described this as a process or journey that involved a combination of actively seeking out knowledge, finding fit, maintaining hope, developing confidence through clinical reasoning, and working on and within the environment.

A number of parallels can be drawn between the occupational therapists' ongoing journey of developing and sustaining recovery-orientation and some aspects of the mental health recovery journey described by consumers. First, both are individually driven [2]. Second, rather than being a singular event, both are characterised as a continual process with no fixed end-point or final destination [3]. Third, each process can be facilitated or hindered by the human and structural elements within the environment [6]. For example, just as participants in this study highlighted the importance of the support and guidance they gave to and received from colleagues, consumers have repeatedly identified the value of giving and receiving peer support from others travelling a similar journey [5]. Fourth, for therapists, strengthening their knowledge, hope, and confidence was essential in cultivating their recovery-oriented practice. Similarly, consumers have described an ongoing need to learn about themselves and make sense of their experiences in order to progress along their recovery journey [2]. Both describe needing hope and a belief that recovery can occur to inspire their ongoing efforts [3]. While consumers describe benefiting from the support of recovery-focused professionals, our participants valued the contributions of recovery-focused consumers to their practice. Perhaps recognition by both occupational therapists and consumers of the parallel journeys being undertaken could facilitate a consciously reciprocal alliance, each mutually supporting the other in their continual efforts and progress.

Participants in this study all described good synergy between principles of recovery-orientation and their occupational therapy professional identity. In this regard, clinicians' experiences are in keeping with perspectives previously expressed by occupational therapy scholars [11, 12, 14], adding credence to this argument. The overwhelming majority of participants in this study, however, described a need to actively find fit, suggesting that the common perception of occupational therapy and recovery-oriented practice as unproblematically synergistic may be an oversimplification. Participants described needing to unlearn aspects of their training and struggling to relinquish their expert role to better embrace principles such as choice and dignity of risk. Indeed, Lal [34] has warned against an oversimplistic assumption that there is perfect symmetry between occupational therapy philosophies and recovery-oriented practice.

Interestingly, while participants' conceptualisations of recovery were mostly consistent with consumer definitions [1], a number included an additional element: independent functioning. This is somewhat in contrast to consumer definitions of recovery in which independence may or may not be an individual's goal. Further, previous research has identified that sense of self and connecting with others, rather than independent functioning, were aspects of occupational engagement that consumers described as most meaningful [35]. The discrepancy is likely due to participants viewing recovery-oriented practice through a traditional occupational therapy lens that prioritises independent function. Similarly, it is meaningful engagement in activities that has been described as critical for recovery rather than the outcomes or products of activities which occupational therapists may be inclined to focus on [13]. Recognising these discipline based values and being wary of inadvertently superimposing them on consumers are important for occupational therapists in developing and sustaining their recovery-oriented practice [13, 34, 36].

Despite the perceived congruence between occupational therapy and recovery compared to other disciplines, participants experienced environmental barriers to and facilitators of recovery-oriented practice that were consistent with findings in previous multidisciplinary research. These included human aspects such as coworker attitudes towards recovery [8, 27, 28] and structural elements such as restrictive contexts [25] and dominance of systemic priorities [26]. Most participants in this study, however, did not merely recognise the environmental influences on their practice; they also described working to exert influence by doing things like using recovery-oriented language and leading by example, defending their practices with strong clinical reasoning, and supporting recovery-orientation in their supervision of others.

However, it is clear that changes at a systemic level are also needed. For example, health services should consider review and modification of KPIs to better align with recovery-oriented principles. Examples of recovery-oriented KPIs might include the percentage of consumers with a wellness or recovery action plan, scores of consumer satisfaction with service, measures of hopefulness, or percentage of consumers with self-identified goals. Health services can also better support therapists to use strategies they have identified to enhance their recovery-orientation. Our research supports the inclusion of peer workers in services and the creation of opportunities for therapists, particularly those working in restrictive or acute environments, to meet and learn from consumers who are further along their recovery journeys. For example, provision of consumer-facilitated training or rotating work positions that enable all therapists to experience working with consumers at varying stages of recovery might facilitate therapists' belief in and hope for recovery and thus their recovery-oriented practice. Because our results suggest that different practice contexts may present unique challenges, a one-size-fits-all, whole of system approach alone may be inadequate in supporting the realisation of recovery-oriented reform. Rather, engagement and troubleshooting at a context-specific level may prove essential to facilitating practice change.

Occupational therapists have been criticised for being “complicit in perpetrating oppressive institutional practices” and falling victim to organisational red tape rather than advocating for consumers [36]. The active strategies described by our participants suggest that occupational therapists may be beginning to take on a leadership role in helping shift mental health practice towards a more recovery-oriented approach as they have been called to do [12–14]. However, participants' spheres of influence appeared to remain at the immediate workplace level. They tried to work creatively within the existing systemic constraints rather than describing advocacy for change at this broader level. The next step for occupational therapy as a profession is to extend our leadership beyond our immediate workplaces and facilitate systemic transformation towards a more recovery-oriented mental health system. This can occur at all levels within the profession. Occupational therapists working directly with consumers can, for example, pursue opportunities to sit in on decision-making committees or panels with influence as well as advocating for consumers to be included on these. University educators can enhance the capacity of new graduates by focusing upon intervention at a systemic, rather than just an individual, level. Occupational therapy associations can also play a role through their advocacy and policy-based activities and provision of continuing professional education focused on facilitating system change.

### 4.1. Limitations

As with any qualitative research, the findings of this study are not universally applicable and should be interpreted with reference to the sample characteristics. All participants worked in New South Wales or Victoria, Australia. While the process of developing and sustaining recovery-oriented practice is likely to be relevant for occupational therapists in other geographical locations, as evidenced by the congruence of the findings with international literature, systemic and cultural differences warrant further research. Additionally, participants' experiences were diverse, and participants themselves identified contextual issues such as training, experience, practice context, and roles as influencing these. Research on larger samples would allow a more robust comparison of therapists' experiences and identification of issues that might be especially pertinent to particular groups. The aim to examine how occupational therapists develop and sustain recovery-orientation required targeted recruitment of therapists who identified themselves as being recovery-oriented. Future research exploring the experiences of those who feel unable or are unwilling to adopt recovery-oriented practice will be beneficial in further understanding the lag between policy and practice.

## 5. Conclusion

This small exploratory study describes how occupational therapists consciously and actively develop and sustain recovery-orientation in their mental health practice. It adds to previous multidisciplinary research by (a) illuminating active strategies, which may well be similarly relevant to other health professionals, and (b) highlighting specific tensions between occupational therapy and recovery-oriented practice frameworks and philosophies. This work provides guidance for therapists regarding the ongoing work needed to realise and maintain their own recovery-oriented practice, as well as challenging occupational therapists to extend their influence to broader system-level reform.

## Figures and Tables

**Table 1:** Participant demographics.

Participant	Years since graduation	Current role			Current setting				
		OT-specific	Generic	Clinical supervisor	Acute/inpatient	Community	Forensic	Youth/early psychosis	NGO^†^
Addison^‡^	6	x				x		x	
Anna	14	x			x		x		
Jessica	16	x		x		x			
Chloe^‡^	<1		x						x
Ava^‡^	<1	x				x			
Chris^‡^	10		x	x					
Pat	22		x	x		x		x	
Amelia^‡^	19		x	x		x		x	
Jordan	13	x		x	x		x		
Charlotte	11		x	x		x			
Jamie	<1	x			x	x		x	
Casey	8	x		x	x		x		

^†^Nongovernmental Organisation.

‡ denotes those who participated in follow-up interviews.
